# New Treatment Strategy Using Repetitive Transcranial Magnetic Stimulation for Post-Stroke Aphasia

**DOI:** 10.3390/diagnostics11101853

**Published:** 2021-10-08

**Authors:** Takatoshi Hara, Masahiro Abo

**Affiliations:** Department of Rehabilitaion Medicine, The Jikei University School of Medicine, Tokyo 105-8461, Japan; abo@jikei.ac.jp

**Keywords:** stroke, aphasia, non-invasive brain stimulation, transcranial magnetic stimulation, neuroimaging

## Abstract

Repetitive transcranial magnetic stimulation (rTMS) for post-stroke aphasia (PSA) has been suggested to promote improvement of language function when used in combination with rehabilitation. However, many challenges remain. In some reports examined by category of language function, only naming has good evidence of improvement, and the improvement effect on other language modalities is low. Therefore, it is necessary to establish methods that contribute to the improvement of language functions other than naming. Therapeutic methods for PSA based on the mechanism of rTMS are mainly inhibitory stimulation methods for language homologous areas. However, the mechanisms of these methods are controversial when inferred from the process of recovery of language function. Low-frequency rTMS applied to the right hemisphere has been shown to be effective in the chronic phase of PSA, but recent studies of the recovery process of language function indicate that this method is unclear. Therefore, it has been suggested that evaluating brain activity using neuroimaging contributes to confirming the effect of rTMS on PSA and the elucidation of the mechanism of functional improvement. In addition, neuroimaging-based stimulation methods (imaging-based rTMS) may lead to further improvements in language function. Few studies have examined neuroimaging and imaging-based rTMS in PSA, and further research is required. In addition, the stimulation site and stimulation parameters of rTMS are likely to depend on the time from onset to intervention. However, there are no reports of studies in patients between 90 and 180 days after onset. Therefore, research during this period is required. New stimulation methods, such as multiple target methods and the latest neuroimaging methods, may contribute to the establishment of new knowledge and new treatment methods in this field.

## 1. Introduction

Stroke is the most disabling health condition worldwide in adults, and a substantial proportion of stroke survivors live with aphasia [1]. Post-stroke aphasia (PSA) is an acquired language disorder that can impair some or all modalities of language processing (speech, listening, reading, and writing). PSA can affect an individual’s ability to express or understand language and can impair communication, socialization, and return to work. Due to early physiological repair mechanisms, stroke patients may achieve some spontaneous recovery even in the absence of rehabilitation treatment. However, approximately 40% of these patients have significant aphasia at one year after stroke, and residual symptoms may persist for many years [2,3]. 

The basis of treatment for PSA is speech and language therapy (SLT). SLT can restore some language function; however, recovery is slow, there is difficulty in maintaining function, and the effect size may not be large [4,5]. The course of ultra-long-term aphasia treatment has demonstrated an almost permanent persistence of SLT is necessary to maintain language function [5]. The improvement of aphasia requires structural and functional reorganization of language networks in the cerebral cortex, and recent research on neural-plasticity has indicated repetitive transcranial magnetic stimulation (rTMS) as a new approach in stroke rehabilitation [6,7,8,9]. As described below, some studies have reported the use of rTMS for PSA. However, considering the process of improving language function, questions remain regarding the mechanism of action of rTMS as well as stimulation methods and parameters. In this review, we discuss the latest findings for PSA, topics that remain controversial in this area, and key findings for solutions.

## 2. rTMS Treatment for Aphasia

The rTMS approach uses magnetic energy to induce change in the excitability the underlying brain cortex in a non-invasive fashion and can induce long-lasting neuroplastic changes. TMS produces a time-varying magnetic field that flows perpendicular to the stimulating coil, which then induces electric currents that are generally parallel to the coil in the underlying cortical tissue. Different stimulation frequencies have different effects on the activity of the cerebral cortex, with high-frequency (>5 Hz) stimulation facilitating local neuronal excitability and low-frequency (<1 Hz) stimulation showing inhibitory effects [10,11]. Currently, rTMS is the mainstream stimulation method in clinical applications and has been applied in the field of psychiatric disorders and, especially, to treat depression [12,13].

Changes in neural activity in the cerebral cortex are induced by rTMS, which promotes plasticity. This stimulation facilitates network-related reconstruction in the brain. However, the improvement of aphasia requires reacquisition of language functions, including motor or sensory elements, in each language modality [14]. Therefore, the combination of SLT is essential for rTMS to be successful. The concept of rehabilitation aimed at improving neuroplasticity suggests that SLT combined with rTMS may induce a positive synergistic effect not only for modulation of neural connections but also for functional re-learning [8,14]. In fact, many reports of rTMS for PSA in recent years have used rTMS in combination with SLT (see table in the next section). 

## 3. Evidence of the Use of rTMS for Post-Stroke Aphasia

**Figure 1:**
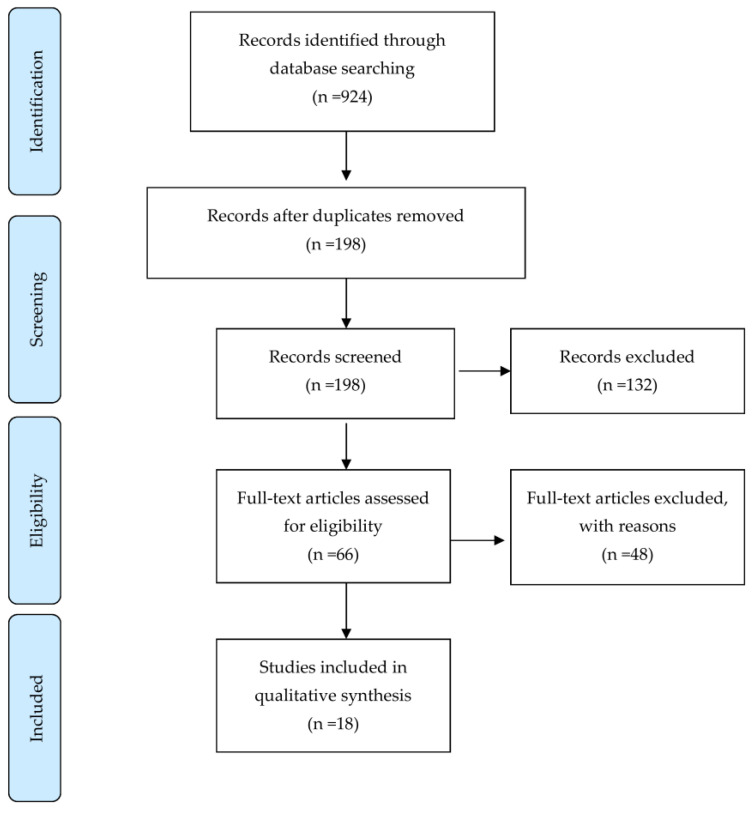
Study flow diagram.

We searched all English articles up to 31 December 2020 using the following databases: Pubmed/MEDLINE, Scopus, CINAHL, and Embase. The following keywords were used in the searches: Stroke, Cerebral vascular accident, Ischemic stroke, Hemorrhagic stroke, Non-invasive brain stimulation, Transcranial magnetic Stimulation, Theta-burst stimulation, Quadripulse stimulation, and Aphasia. Articles reporting on randomized and prospective controlled trials (RCT and PCT, respectively) were included, and case studies were excluded. We identified 198 records through the searches after removal of duplicates. No additional records from other sources were identified. After screening the titles and abstracts, we excluded 132 records mainly because the studies were animal studies, abstracts only, articles reporting on protocols, in-progress trials, retrospective studies or case reports, systematic review, non-English language publications, and completely irrelevant articles. After further assessment, 18 studies were considered to meet the review inclusion criteria (Figure 1). Details of these studies are presented in Table 1.

Changes in neuroplasticity associated with the amelioration of PSA are associated with the principle of intercerebral hemisphere inhibition and the relationship between the activation of the language areas and the activation of the language homologous areas. However, the role of the left and right cerebral hemispheres in the recovery process is still under debate, as described later. Early studies of rTMS for PSA showed that low-frequency rTMS (LF-rTMS) targeting Broca’s area was effective in brain reconstruction and contributed to the improvement of non-fluent aphasia [22,32]. The target of stimulation in many studies is the IFG in the right cerebral hemisphere [34] since the language homologous areas of the right cerebral hemisphere temporarily support language function when the major area of language in the left cerebral hemisphere is damaged. Over time, the language homologous areas are associated with maladaptation of activation of the language field in the left cerebral hemisphere [8].

According to a recent meta-analysis of rTMS treatment for PSA, Shah-Basak et al. extracted five RCTs and four non-RCTs with Standardized Mean Difference (SMD) = 0.448 (95% CI = 0.23–0.66) [35]. Similarly, Bucur et al. extracted eight rTMS studies in a systematic review focusing on naming performance and indicated that SMD = 0.71 (95% CI = 0.43–1.00), with examining at the time of intervention from the onset, SMD = 0.62 (95% CI = 0.25–0.99) in the chronic phase and SMD = 0.85 (95% CI = 0.38–1.32) in the subacute phase [36]. However, for the subacute phase, only three studies were extracted, and they tended to be highly heterogeneous (I2 = 15.2%). According to the subanalysis, the effect in the chronic phase was 0.62 (95% CI = 0.25–0.98) in weighted mean effect sizes from five studies.

On the other hand, some reports have mentioned that the effect is limited. A meta-analysis that extracted LF-TMS, HF-rTMS, and bilateral-rTMS only had a significant effect on naming performance and no significant effect on other language modalities [37]. Kim et al. also evaluated the quality of evidence using the Grade of Recommendation, Assessment, Development, and Evaluation (GRADE) tool and found low quality (i.e., further research is very likely to have an important impact on our confidence in the estimate of effect and is likely to change the estimate) [38]. Therefore, it is suggested that the evidence of the effect of rTMS on PSA is limited, and evidence is limited to the chronic phase. In fact, a review of rTMS treatment for various diseases by Lefaucheur et al. concluded that rTMS for PSA is “probable efficacy of LF-rTMS of right IFG in nonfluent aphasia recovery at the chronic stage (Level B)” [34]. Therefore, further accumulation of research is required, and it is necessary to verify the effects classified by time from onset and by language modality other than naming performance.

## 4. Relationship between rTMS and Language Function Recovery in PSA

Kolb’s concept of cerebral plasticity began to gain attention in regards to aphasia in the 1980s and suggests that the right hemisphere could take over the major language functions of the left hemisphere [39]. With the development of imaging techniques, studies on the recovery process of language function using functional neuroimaging techniques (PET and fMRI) have been reported, and further focus has been placed on activation of the right cerebral hemisphere and the results of language recovery for PSA [40,41,42]. When initial research on magnetic stimulation for PSA was first reported, right hemisphere areas were suggested to support some language recovery only if essential language areas of the left hemisphere are destroyed [43]. In addition, Saur et al. noted that transient activation of right hemisphere networks may be necessary to achieve good recovery and normalization of left hemisphere network activity [44]. It was inferred that the language homologous areas complement some language function after a stroke. On the other hand, Postman-Caucheteux et al. suggested that greater damage to the left hemisphere induces more involvement of the right hemisphere and poorer functional language recovery [40]. Richter et al. reported no correlation between language improvement and right hemispheric activation in subjects with aphasia [41]. Thus, the activation of the language homologous areas in the right cerebral hemisphere does not necessarily indicate the complementation of language function, suggesting the possibility of “reactive activation” or “inefficient activation”. In addition, it is necessary to consider that the role of activation in these right cerebral hemispheres changes significantly depending on the recovery process over time from the onset of stroke.

In rTMS therapy for post-stroke motor function, low-frequency rTMS of the contralesional hemisphere is recommended based on the theory of interhemispheric inhibition [34]. In the motor system, transcallosal inhibitory connections between the primary motor cortices of the two hemispheres may help to coordinate bimanual movement [45]. Normally, suppression between the cerebral hemispheres is similar. However, the onset of stroke reduces suppression from the lesioned hemisphere to the contralesional hemisphere, which strengthens suppression from the contralesional hemisphere side to the lesioned hemisphere. Thus, it may become difficult to induce an improvement in the plasticity of the area around the injury, which is important for recovery after stroke [46]. This inhibitory imbalance between hemispheres inhibits functional recovery, and inhibition of the contralesional motor cortex using rTMS enhances cortical excitability on the lesion side [47,48,49]. In application of this hypothesis of interhemispheric inhibition to language function, Naeser et al. performed 1-Hz inhibitory stimulation on the language homologous site of the right cerebral hemisphere and reported improvement in language function in four non-fluency aphasia patients [50]. The effectiveness of rTMS therapy for PSA in these studies suggests a therapeutic protocol based on interhemispheric inhibition [32,51,52].

## 5. Selection of Stimulation Site Inferred from the Process of Improving Language Function

**Figure 2:**
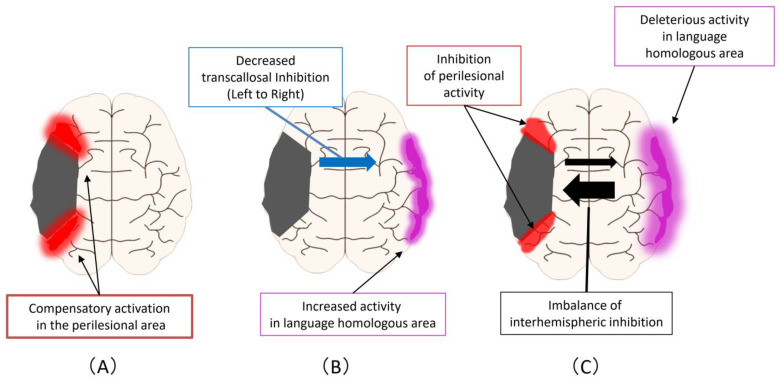
Improvement mechanism for language function in PSA: (**A**) Compensatory activations in the perilesional area, that can occur at any of the acute, subacute, and chronic phases; (**B**) As the next step, an enhancement of activity occurs at homologous areas in the language domain. This reaction is triggered by a decreased transcallosal inhibition from the ipsilesional hemisphere to the contralesional hemisphere that can occur during the acute or subacute phase; (**C**) As the next step, an imbalance of interhemispheric inhibition forms, accompanied by activation of homologous areas in the language region and impediments to functional recovery in the perilesional area.

Figure 2 shows the improvement mechanism for language function in PSA. First, there are compensatory activations in the perilesional area that can occur at any of the acute, subacute, and chronic phases (Figure 2A) Second, an enhancement of activity occurs at homologous areas in the language domain, which may complement primary language function areas in the ipsilesional hemisphere (Figure 2B). This reaction is triggered by a decreased transcallosal inhibition from the ipsilesional hemisphere to the contralesional hemisphere that can occur during the acute or subacute phase. Third, an imbalance of interhemispheric inhibition forms, accompanied by activation of homologous areas in the language region and impediments to functional recovery in the perilesional area (Figure 2C). This reaction is a change that occurs mostly in the chronic phase.

**Figure 3:**
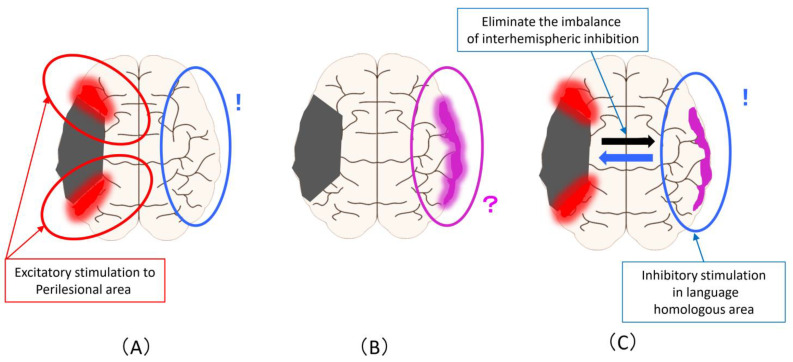
Magnetic stimulation site that can be assumed from the improvement mechanism related to language function in PSA: (**A**) Excitatory stimulation of the ipsilesional areas to promote compensatory activation in the perilesional areas (Red circle). the inhibitory stimulation to the language homologous areas based on the principle of interhemispheric inhibition (Blue circle); (**B**) Regarding the increase in activity in the homologous areas of the language domain, the stimulation site and stimulation methods are unclear (Pink circle); (**C**) An inhibitory stimulation to the language homology areas that eliminates the imbalance of interhe-mispheric inhibition and reduce the activity of the language homology areas (Blue circle).

Figure 3 shows the magnetic stimulation site that can be proposed from the changes described in Figure 1. Excitatory stimulation of the ipsilesional areas results in a more advanced promotion of compensatory activation in the perilesional areas (Figure 3A). Alternatively, it is the inhibitory stimulation to the language homologous areas based on the principle of interhemispheric inhibition. Regarding the increase in activity in the homologous areas of the language domain, it is not possible to judge whether this activity complements or inhibits the activity in the language area, so the stimulation site and stimulation methods are controversial (Figure 3B). To solve this problem, it is necessary to clarify it from the evidence of rTMS and brain function imaging in the subacute phase (as is described later). It is an inhibitory stimulation to the language homology areas that eliminates the imbalance of interhemispheric inhibition, that is, to reduce the activity of the language homology areas (Figure 3C).

From Figure 3, the biggest problem is that the inhibitory stimulation to language homologous areas performed based on the principle of interhemispheric inhibition (Figure 3A) and inhibitory stimulation performed for the purpose of eliminating overactivity of language homologous areas or imbalances of interhemispheric inhibition (Figure 3C) are the same method despite the difference in the assumed mechanism of action and the background activity of the cerebral hemisphere. In other words, the most evidence-proven method of stimulating the language homologous areas of the contralesional hemisphere may have been performed under a mixed background and an unknown mechanism of action.

## 6. Questions about Language Recovery Processes and Stimulation Sites Associated with rTMS

Some researchers suggest that there may be challenges in inhibitory stimulation of the right cerebral hemisphere language homology areas. A study by de Mendonca cites the following three points as questions [53]: (1) Is inhibition of the right hemisphere truly beneficial?; (2) Is the transference of the language network to the left hemisphere truly desirable in all patients?; and (3) Is the use of TMS during the post-stroke subacute phase truly appropriate? Right hemisphere inhibition has been the most commonly used treatment strategy to date, after the pioneering work of Naeser et al. [50], but this consistency may be misleading [54]. In addition, as of 2006, Heiss et al. focused on the extent of damage [43]. In the case of a widespread lesion in the middle cerebral artery (MCA) region, even if an inhibitory stimulation is applied to the right cerebral hemisphere, if there is no room to activate the left cerebral hemisphere, it is possible that rTMS would have no effect, with no improvement in language function [43]. Khedr et al. also suggested the need for high-frequency magnetic stimulation of the right hemisphere, as the effect of rTMS on only one hemisphere is inadequate for patients with complete infarction in the left MCA region [23]. Therefore, further research is needed to address these questions.

Some reports have suggested that the language homologous part of the right cerebral hemisphere is not a temporary functional complement in the process of recovery of PSA language function. Winhuisen et al. identified a language-activated region based on PET in the acute phase and performed inhibitory stimulation to that region, a stimulation method that impairs language function [55], and their results showed that inhibitory stimulation led to impaired verb production [55]. Thiel et al. reported similar findings in cases of right-handed patients with left hemisphere brain tumors [56]. In addition, Turkeltaub et al., in a case report, showed that inhibitory stimulation to the right IFG improved language function; however, three months later, the patient had a right hemisphere cerebral infarction that exacerbated the aphasia [57]. The role of these right cerebral hemispheres is unclear, and those results may be related to the passage of time from recovery. Anglade et al. proposed the following from the role of the right cerebral hemisphere in the process of recovery of language function, focusing on the size of the injured area [58]:

1. Concerning limited linguistic impairments with good anatomic preservation of the primary language-processing areas (Broca and Wernicke areas), there is a good prognosis for language recovery with weak right cerebral activation. This setting could be described as the optimal recovery case.

2. Moderate linguistic impairments with a more extended but incomplete lesion of these same areas are associated with a stronger right cerebral activation during the subacute phase and a shift back towards the left hemisphere during the chronic phase. This scenario is associated with greater linguistic recovery and can be described as a partial recovery case, and individuals could benefit from cerebral stimulation aimed at inhibiting the right hemisphere.

3. Severe linguistic impairments with near complete destruction of the primary language-processing areas (large left infarct) involve a significant right hemisphere activation during the sub-acute and chronic phase. The behavioral results demonstrate only a very moderate functional improvement, considerably less than with other recovery patterns, and only the maintenance of low-level automatic speech can be expected.

Theories based on these damaged areas are consistent. Therefore, it is necessary to investigate these inferences as well as new theories and methods to solve these remaining questions.

## 7. Imaging-Based rTMS for PSA

Kakuda et al. performed a repeat task using functional MRI in patients with motor aphasia, identified the brain activation area, and performed a method of stimulating the contralateral site corresponding to the homologous site [59]. Low-frequency rTMS performed on four cases showed improvement in language function in all cases. Similarly, Abo et al. classified non-fluent aphasia and fluent aphasia based on a repeat task using functional MRI and performed low-frequency rTMS and intensive speech therapy to activate the identified activation sites [60]. An 11-day protocol was performed, with significant improvement in the Western Aphasia Battery (WAB) in the fluent aphasia group between before and after the intervention. In comparison with one month after the intervention, the Standard Language Test of Aphasia (SLTA) showed significant improvement in language function in both the non-fluent aphasia group and the fluent aphasia group. In the sub-item analysis, a significant improvement in spontaneous speech was observed in the non-fluent aphasia group, and a significant improvement in auditory comprehension and reading comprehension was observed in the fluent aphasia group.

From these past reports, we clarified the role of the right cerebral hemisphere, which is affected by the time course of recovery from onset, by identifying the activated region using functional MRI during the process of recovery of language function. We speculated that it would be possible to induce improvement in language function as well. In addition, we determined whether there was a change in cerebral blood flow, as inferred before and after the intervention, by the following method [8]. Based on functional MRI, the patients were classified into a low-frequency rTMS group for the contralesional hemisphere and a low-frequency rTMS group for the ipsilesional hemisphere, and changes in the regional cerebral blood flow (rCBF) before and after the intervention were assessed by Single Photon Emission Computed Tomography (SPECT). The values of rCBF in 14 regions, which are language-related regions, were calculated, and the laterality index (LI) was calculated. The relationship between the changes before and after the LI intervention (ΔLIs) and the SLTA before and after intervention was statistically analyzed. The SLTA total mean score improved from 148.8 to 154.7 and 127.0 to 133.6 in the RH-LF-rTMS and LH-LF-rTMS groups (*p* < 0.01), respectively. Correlation analyses between the SLTA total change scores and rCBF ΔLIs showed a statistically significant association in BA44 in the RH-LF-rTMS group (*r* = 0.402, *p* < 0.05, R^2^ = 0.144). However, the LH-LF-rTMS group did not show any significant association between the SLTA total change scores and rCBF ΔLIs. In terms of the sub-items of the SLTA, SLTA subscale change scores and rCBF ΔLIs were examined in the RH-LF-rTMS group, and statistically significant associations were detected in BA11, 20, and 21 for the speaking subscale and in BA6 and 39 for the writing subscale. In the LH-LF-rTMS group, significant associations were observed in BA10 for the speaking subscale and in BA13, 20, 22, and 24 for the reading subscale.

There were some interesting points in this study. First, in the two groups of SLTA divided by functional MRI before intervention to identify the language activation region, LH-LF-rTMS was lower than RH-LF-rTMS at the baseline of total SLTA. This indicates that the proportion of patients with severe language impairment was high in the LH-LF-rTMS group, which was consistent with the above-mentioned reasoning by Anglade et al. [58]. In other words, individuals in the LH-LF-rTMS group may not have a shift in language function activity from the contralesional hemisphere (right cerebral hemisphere) to the ipsilesional hemisphere (left cerebral hemisphere). Second, we identified significant improvement in language function; however, only RH-LF-rTMS and not LH-LF-rTMS was significantly correlated with changes before and after total SLTA intervention. In the sub-items, the LH-LF-rTMS group had a significant correlation with ΔLIs in reading, but no significant improvement in scores was observed before and after the intervention in this item. In other words, low-frequency rTMS for the contralesional hemisphere showed a significant improvement in language function, as in previous reports. On the other hand, although low-frequency rTMS and intensive speech therapy for the ipsilesional hemisphere showed significant improvement in speech function, it was suggested that the effect may be limited. Therefore, it is necessary to establish an effective rTMS method for patients with significant activation of the right cerebral hemisphere.

To solve this issue, our research group reexamined the methods used [61]. Using functional near-infrared spectroscopy (fNIRS), we performed a repeat task, as in previous studies, and identified language-activated regions. For the left cerebral hemisphere activation case, we adopted low-frequency rTMS for language homology areas as before (RH-LF-rTMS group). However, in the case of activation of the right cerebral hemisphere, high-frequency rTMS for language homology sites was adopted (RH-HF-rTMS group). In cases where the right cerebral hemisphere was activated, activation of the perilesional areas may not be expected due to lesions in the left wide area or major language area. This method, combined with intensive speech therapy, showed a significant improvement in SLTA in both groups. Analysis of changes in rCBF of fNIRS before and after the intervention showed that, in the RH-LF-rTMS group, activation just below the magnetic stimulation site in the right cerebral hemisphere decreased, and activation of the language field in the left cerebral hemisphere became more localized. On the other hand, in the RH-HF-rTMS group, activation directly under magnetic stimulation in the right cerebral hemisphere was further increased.

This result shows that, in the previous method, the improvement of language function and the change of cerebral blood flow were inconsistent, but in this recent method, the improvement of language function and the improvement of cerebral blood flow changed in parallel in both groups. This is consistent with high-frequency adoption in the right cerebral hemisphere mentioned by Khedr et al. [23]. In the present study, we did not verify the direct relationship between low-frequency stimulation to the ipsilesional site and high-frequency stimulation to the contralesional side for activated cases of language homologous areas based on fNIRS. Therefore, in the future, it will be necessary to verify two types of stimulation patterns for activated cases of language homologous areas. 

## 8. rTMS and Neuroimaging Study for PSA

**Figure 4:**
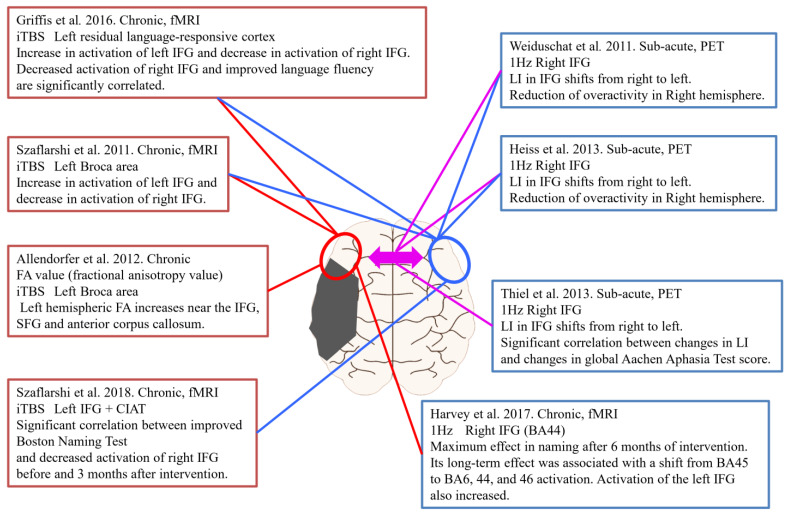
rTMS intervention and Neuroimaging assessment in PSA.

Assessing changes in brain activity before and after rTMS, including our study above, is useful not only for therapeutic effects but also for determining the effectiveness of stimulation sites and stimulation methods. Figure 4 shows neuroimaging studies before and after rTMS for PSA. Four studies used intermittent theta burst stimulation (iTBS), and the remaining four studies used low-frequency rTMS. Five of the studies were performed in the chronic phase, and the remaining three studies were performed in the subacute phase. In addition, all studies in the chronic phase used fMRI to evaluate brain function activity before and after the intervention. On the other hand, the three studies in the subacute stages were evaluated using PET.

Szaflarshi et al. reported increased activation of the left fronto-temporo-parietal language networks and decreased activation of the right language homologous site by fMRI with iTBS stimulation of the left Broca’s area in patients with chronic PSA [62]. Similarly, Griffis et al. stimulated the left language residual region with iTBS and reported an increase in left IFG activation and a decrease in right IFG activation on fMRI. In addition, there was a significant correlation between decreased activation of the right IFG and improved fluency in aphasia [63]. Allendorfer et al. used MRI to calculate fractional anisotropy value (FA), and iTBS stimulation targeting the left Broca area showed an increase in FA in the left inferior and superior frontal gyri and anterior corpus callosum [64]. In a study of iTBS stimulation combined with constraint-induced aphasia therapy (CIAT), there was a significant correlation between an improved Boston Naming Test and decreased activation of the right IFG before and at three months after intervention in the evaluation of brain activity using fMRI [65]. Harvey et al. performed 1-Hz low-frequency rTMS on the right IFG and evaluated changes in brain activation using fMRI [66]. According to that study, language function was most effective in naming after six months. Its long-term effect was associated with the transition of activation of brain activity from BA45 to BA6, 44, and 46. In addition, activation of the left IFG also increased. In a study using low-frequency rTMS targeting the right IFG in the subacute phase, PET was used to evaluate brain function before and after the intervention, with a shift of LI from right to left in IFG and overactivity of the right cerebral hemisphere [27,52]. Similarly, Thiel et al. reported that, in addition to the right-to-left shift of LI, changes in LI and changes in language function evaluation scores showed a significant correlation [25]. Together, these results suggest that evaluating changes in cerebral blood flow and brain function activity in parallel with the effect of PSA on language function in the intervention of rTMS can be expected to strengthen the evidence in choice of stimulation site and stimulation parameters. In other words, further research using the above-mentioned “imaging-based rTMS” method is needed.

## 9. Stimulation Site of rTMS Considered from the Time of Onset

As mentioned above, in the process of PSA recovery from onset to stimulation, plasticity changes occur in the cerebral hemisphere for improvement. For this reason, each language modality has different areas of activity, but it is important to further reinforce the brain activity that supports their recovery.

Regarding time between stroke onset and treatment, if 30 days or less is classified as acute phase, 30 days to 180 days is classified as subacute phase, and 6 months or later is classified as chronic phase. The relationship between past RCT and PCT reports and the stimulation site is shown in Table 2. The subacute phase was divided into two periods: 30 to 90 days and 90 to 180 days. In the report on the acute phase, the study of Waldowski et al. was classified during the acute phase [30]. In the subacute phase (30 to 90 days), seven studies were classified [16,18,19,23,25,26,27]. In the study by Heiss et al., the stimulation site was different between right-handed and left-handed individuals [27]. The study by Khedr et al. is the only study that employed bilateral stimulation in the subacute phase [23]. On the other hand, none of the studies corresponded to the subacute phase of 90 to 180 days). Ten studies were classified in the chronic phase [15,17,20,21,22,24,28,29,31,32,33]. Of these, Ren et al. selected two stimulation patterns, Rt IFG and Rt STG. In addition, there were two studies in which neuroimaging was performed, both of which were subacute (30 to 90 days) studies (Figure 3) [25,27].

As mentioned above, it is important to use neuroimaging to strengthen the evidence of rTMS for PSA. However, in the past research using RCT and PCT, there were only two studies. Therefore, further research using the imaging-based rTMS method is required in the future. Many of the rTMS studies in the subacute phase include patients at approximately 30 days after onset. These cases are close to the acute phase, and from the viewpoint of the process of recovery of language function from the onset, the activity of the right cerebral hemisphere is imminent in these cases. Therefore, stimulation of the right IFG in these cases aims to activate the activity of the perilesional areas in the left cerebral hemisphere with a remote effect, mainly through the network between hemispheres (Figure 2A). On the other hand, it can be seen that there is no study in Figure 2B at the time when the increase in activity in the homologous part of the language domain is expected to appear. As mentioned above, the increase in activity in the homologous part of the language domain is controversial because it cannot be determined whether this activity complements or inhibits the activity in the language domain. For this reason, the selection of only one stimulation site may be difficult. Therefore, bilateral rTMS may be an effective method, as in the study by Khedr et al [23]. Actually, the multiple-target method was proposed as a possible effective approach as a treatment for cognitive dysfunction and psychiatric disorders after stroke [14,67,68,69]. 

## 10. Conclusions

Studies have suggested that rTMS for PSA may promote improvement of language function when used in combination with rehabilitation. However, many challenges in the use of these methods remain:

1. In some reports that examined the effects of rTMS by category of language function, only naming shows good evidence of an effect, and the improvement effect on other language modalities is not high. Therefore, it is necessary to establish methods that contribute to the improvement of language functions other than naming. 

2. Therapeutic methods for PSA based on the mechanism of rTMS are mainly inhibitory stimulation methods for language homologous areas. However, the mechanism of this method is still controversial when inferred from the process of recovery of language function. 

3. Low-frequency rTMS for the right hemisphere has been shown to be effective in the chronic phase, but recent studies of the recovery process of language function indicate that this mechanism is unclear. Therefore, evaluating brain activity using neuroimaging contributes to clarification of the mechanism of rTMS on PSA and the elucidation of the mechanism of functional improvement. 

4. Neuroimaging-based stimulation methods (imaging-based rTMS) may lead to further improvements in language function. There are still few studies on neuroimaging and imaging-based rTMS in PSA, and further research is required. 

5. The stimulation site and stimulation parameters of rTMS are likely to depend on the time from onset to intervention. However, there are no reports of studies in patients between 90 and 180 days after onset. Therefore, research during this period is required. 

New stimulation methods, such as multiple target methods and the latest neuroimaging methods, may contribute to the establishment of new knowledge and new treatment methods in this field. 

## Figures and Tables

**Table 1:** Individual Study Characteristics, Treatment Characteristics, Assessments, and Outcomes.

Study	Design	Sample/ Age (SD)	Time between Stroke Onset and Treatment	Stimulation Site	Parameter /Session	Speech Training	Assessments	Follow-Up	Results
Ren et al. 2019 [15]	RCT IFG group or STG group or Sham	18,18,18/ 65.95 (8.53), 62.46 (10.95), 63.60 (16.71)	55.90 (19.41), 50.58 (23.80), 61.20 (22.66) days	Right posterior IFG or Right posterior STG	1 Hz 80% MT 1200 pulses/session 3 weeks	30 min per day. Specific training of specific language features.	WAB	3 weeks after stimulation	Compared with the sham group, the increases were significant for auditory comprehension, repetition, and AQ in the pSTG group (*p* < 0.05), whereas the changes in repetition, spontaneous speech, and AQ tended to be higher in the pIFG group.
Heikkinen et al. 2019 [16]	RCT rTMS or Placebo	17/ 54 (9.94), 61 (7.47)	34 (490.77), 48 (881.69) months	Right IFG	1 Hz 90% MT 1200 pulses/session 10 sessions	3 h/day for a total of 10 days. Naming. Intensive Language-Action Therapy (ILAT)	WAB, BNT, ANT	4, 7 weeks, and 3 months after stimulation	ILAT was associated with significant improvement across groups. No significant effect of rTMS.
Hu et al. 2018 [17]	Divided randomly into four groups (HF group or LF group or Sham group or Control group)	40/ 46.5 (12.1), 48.5 (11.2), 50.7 (10.4), 47.3 (9.8)	7.1 ( 2.7), 7.5 (3.2), 6.8 (2.3), 7.7 (3.4) months	Right IFG	1 Hz or 10 Hz 80% MT 600 pulses/ session 10 sessions	30 min per day. Naming of objects, pictures and scenes.	WAB with Aphasia Quotient	2 months after stimulation	When measured immediately post treatment as well as at 2 months post treatment, the LF group exhibited a more marked improvement than the HF group in spontaneous speech, auditory comprehension. Compared to the control group, the HF group exhibited significant improvement at 2 months post treatment in repetition.
Haghighi et al. 2017 [18]	RCT rTMS group or Sham group	12/ 55 years	subacute (1 month after stroke)	Right IFG	1 Hz 100% MT 1200 pulses/session 10 sessions	45 min per day. Work on individual linguistic symptoms and linguistic deficits.	WAB with Aphasia Quotient	N/A	Speech and language improved over tim, but more so in the rTMS group than in the sham condition. Large effect sizes were observed for content, fluency, and the aphasia quotient; medium effect sizes were observed for command comprehension and repetition, while in auditory comprehension and naming, effect sizes were small.
Rubi-Fessen et al. 2015 [19]	RCT rTMS group or Sham group	19/ 67.9 (8.12), 69.60 (6.67)	41.47 (21.51), 48.73 (21.57) days	Right IFG	1 Hz 90% MT 1200 pulses/session 10 sessions	Oral naming.	AAT, Naming screening ANELT	N/A	The rTMS group significantly improved with respect to all 10 measures of basic linguistic skills, whereas sham group significantly improved in only 6 of 10 measures. There was a significant difference in the gains made by the 2 groups on 5 of 10 measures, including functional communication in ANELT.
Yoon et al. 2015 [20]	PCT rTMS group or Control group	20/ 60.46 (9.63), 61.13 (8.72)	6.80 (2.39), 5.20 (2.67) months	Right IFG	1 Hz 90% MT 1200 pulses/session 20 sessions	60 min, twice a week, for 4 weeks. Conventional SLT	WAB	N/A	Significant improvements in repetition and naming in the rTMS group, but no significant improvement was noted in control group.
Wang et al. 2014 [21]	RCT rTMS underwent synchronous picture-naming training group or rTMS after picture-naming training group, or Sham underwent synchronous picture-naming training group	45/ 61.3 (13.2), 62.1 (12.7), 60.4 (11.9)	16.8 (6.4), 15.7 (8.5), 16.1 (7.3) months	Right IFG	1 Hz 90% MT 1200 pulses/session 10 sessions	60 min, twice a week. SLT about verbal expressive skills	CCAT	3 months after stimulation	rTMS with synchronous picture-naming training group showed significantly superior results in CCAT, expression and description subtests, and action- and object-naming activity. The superior results lasted for 3 months in comparison with the rTMS after picture-naming training group and sham with synchronous picture-naming training group.
Tsai et al. 2014 [22]	RCT rTMS group or Sham group	56/ 62.3 (12.1), 62.8 (14.5)	17.8 (7.2), 18.3 (8.2) months	Right IFG	1 Hz 90% MT 600 pulses/session 10 sessions	60 min. Expression production	CCAT	3 months after stimulation	The rTMS group showed significantly greater improvement than the sham group in CCAT scoring, object-naming accuracy, and naming reaction time. The CCAT scoring and naming testing changes for the rTMS group were persistent at 3 months following intervention.
Khedr et al. 2014 [23]	RCT rTMS group or Sham group	30/ 61.0 (9.8), 57.4 (9.6)	5.8 (4.08), 4.0 (2.6) weeks	Bilateral IFG	Right IFG: 1 Hz 110% MT 1000 pulses/session 10 sessions Left IFG 20 Hz 80% MT 5 s/trains 1000 pulses/session 10 sessons	30 min. SLT using subtests of BDAE	HSS language score, ASRS	2 months after stimulation	There was a significantly greater improvement in the HSS language score after rTMS compared with sham group, which remained significant 2 months after the end of the treatment sessions.
Chieffo et al. 2014 [24]	RCT Patients received 1 Hz, 10 Hz and Sham rTMS. Three sessions for each patient separated by a 6-day washout period.	5/ 54.8 (8.4)	3.2 (1.6) years	Right IFG	1 Hz 100% MT 900 pulses/session 10 Hz 100% MT 800 pulses/session (15 min) Each stimulation was 3 sessions	No training	AAT and Snodgrass naming test.	N/A	10 Hz rTMS was associated with a significant improvement in naming performance and was significantly more effective than 1 Hz rTMS.
Thiel et al. 2013 [25]	RCT rTMS group or Sham group	30/ 69.8 (7.96), 71.2 (7.78)	37.5 (18.52), 50.6 (22.63) days	Right IFG	1 Hz 90% MT 1200 pulses/session 15 sessions	45 min. Deficit-specific aphasia therapy focused on individual linguistic symptoms	AAT	N/A	The change of AAT was significantly higher in the rTMS group. Increases were largest for subtest naming and tended to be higher for comprehension, token test, and writing.
Seniów et al. 2013 [26]	RCT rTMS group or Sham group	40/ 61.8 (11.8), 59.7 (10.7)	33.5 (24.1), 39.9 (28.9) days	Right IFG	1 Hz 90% MT 1800 pulses/session 15 sessions	45 min. Individual linguistic symptoms, expression, and comprehension of spoken language.	BDAE	15 weeks	Language functions improved in both groups after 3 weeks, but only slight group differences in degree of recovery were revealed between patients receiving rTMS and control participants. In repetition, follow-up revealed that severely aphasic rTMS group demonstrated significantly greater improvement than the sham group.
Heiss et al. 2013 [27]	RCT rTMS group or Sham group (left-handed patients received rTMS only)	29 right-handed (+2 left-handed)/ Right 69.0 (6.33), 68.5 (8.19) Left 64, 72	Right-handed 50.1 (23.96) 39.7 (18.43) days Left-handed 25, 93 days	Right-handed: Right IFG Left-handed: Left IFG	1 Hz 90% MT 1200 pulses/session 10 sessions	45 min. Activated networks in the dominant hemisphere	AAT	N/A	Right-handed patients treated with rTMS showed better recovery of language function in AAT as well as in picture-naming performance than sham group. Both left-handed patients also improved in AAT.
Barwood et al. 2013 [28]	RCT rTMS group or Placebo group	12/ 63.7 (7.9)	3.6 (1.3) years	Right IFG	1 Hz 90% MT 1200 pulses/session 10 sessions	No training	BNT, subsets of BDAE, and Snodgrass and Vanderwart naming test.	2, 8, and 12 motnths after stimulation	Significant changes were observed up to 12 months post stimulation in naming performance, language expression, and auditory comprehension in the rTMS group compared to the placebo group.
Medina et al. 2012 [29]	RCT rTMS group or Sham group	10/ 61.60 (8.32)	Chronic (>6 months)	Right IFG	1 Hz 90% MT 1200 pulses/session 10 sessions	No training	Cookie Theft Picture Description of the BDAE and naming tasks.	2 months after stimulation	Across all subjects, the rTMS group resulted in a significant increase in multiple measures of discourse productivity compared to baseline performance. There was no significant increase in measures of sentence productivity or grammatical accuracy. There was no significant increase from baseline in the sham condition on any study measures.
Waldowski et al. 2012 [30]	RCT rTMS group or Sham group	26/ 62.31 (11.03), 60.15 (10.58)	28.92 (19.39) 48.54 (32.33) days	Right IFG	1 Hz 90% MT 1800 pulses/session 15 sessions	45 min. Focused on expression and comprehension of spoken language.	CPNT, BDAE, ASRS	15 weeks	Both groups significantly improved their naming abilities after treatment, but no significant differences were noted between the rTMS and sham groups. The additional analyses have revealed that rTMS subgroup with a lesion, including the anterior part of language area, showed greater improvement primarily in naming reaction time 15 weeks after completion of the therapeutic treatment. Improvement was also demonstrated in functional communication abilities.
Kindler et al. 2012 [31]	RCT and cross over trail TBS group or Sham group	18/ 55.0 (8.6)	15.3 (0.5–40.9) months	Right IFG	30 Hz 90% MT total 801 pulses 3 pulses at 30 Hz 267 continuous bursts 44 s train duration Interval between bursts 100 ms Two sessions on 2 different days separated by 1 week.	No training	A picture-naming task and a language-independent alertness test	N/A	Naming performance was significantly better, and naming latency was significantly shorter after TBS than after sham. Patients who responded best were in the subacute phase after stroke.
Barwood et al. 2011 [32,33]	RCT rTMS group or Placebo group	12 60.8 (5.98), 67 (13.11)	3.49 (1.27) years 3.46 (1.53)	Right IFG	1 Hz 90% MT 1200 pulses/session 10 sessions	No training	BNT, BDAE, CPNT	2 months after stimulation	Significant improvements in naming accuracy, latency, and repetition for the rTMS group compared with sham group. Significant improvements in naming performance, language expression, and auditory comprehension for the rTMS group at 2 months post stimulation.

Abbreviations: AAT, Aachen Aphasia Test; ANELT, Amsterdam-Nijmegen Everyday Language Test; ANT, Action-Naming test; ASRS, Aphasia Severity Rating Scale; BDAE, Boston Diagnostic Aphasia Examination; BNT, Boston Naming test; CCAT, Concise Chinese Aphasia test (CCAT) score; CPNT, Computerized Picture-Naming Test; HSS, Hemispheric Stroke Scale; IFG, inferior frontal gyrus; PCT, pragmatic randomized controlled trial; RCT, randomized controlled trial; SLT, speech language therapy; STG, superior temporal gyrus; TBS, theta burst stimulation; and WAB, Western Aphasia Battery.

**Table 2:** Distribution of previous rTMS studies and the timing of intervention from stroke onset.

Study
Acute
Lt IFG: None Rt IFG: Waldowski et al., 2012 [30] Other: None
Subacute (30–90 days)
Lt IFG: Heiss et al., 2013 (Left handed) [27] Rr IFG: Heikkinen et al., 2019 [16], Haghighi et al., 2018 [18], Rubi-Fessen et al., 2015 [19], Thiel et al., 2013 [25], Seniów et al., 2013 [26], Heiss et al., 2013 (Right handed) [27] Other: Khedr et al., 2014 (Bilateral IFG) [23]
Subacute (90–180 days)
Lt IFG: None Rt IFG: None Other: None
Chronic
Lt IFG: None Rt IFG: Hu et al., 2018 [17], Yoon et al., 2015 [20], Wang et al., 2014 [21], Tsai et al., 2014 [22], Chieffo et al., 2014 [24], Barwood et al., 2013 [28], Medina et al., 2012 [29], Kindler et al., 2012 [31], Barwood et al., 2011 [32,33] Other: Ren et al., 2019 ( Rt IFG or Rt STG) [15]

IFG, inferior frontal gyrus; STG, superior temporal gyrus; Lt, left; Rt, right.
